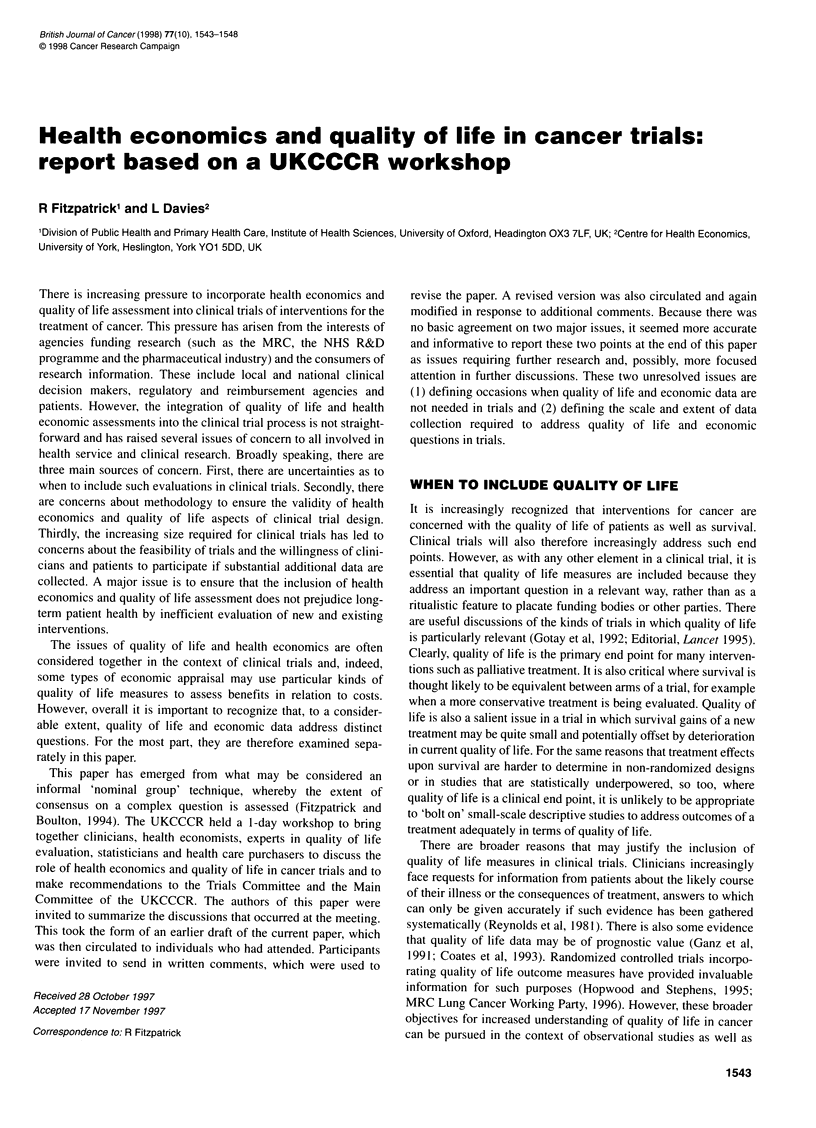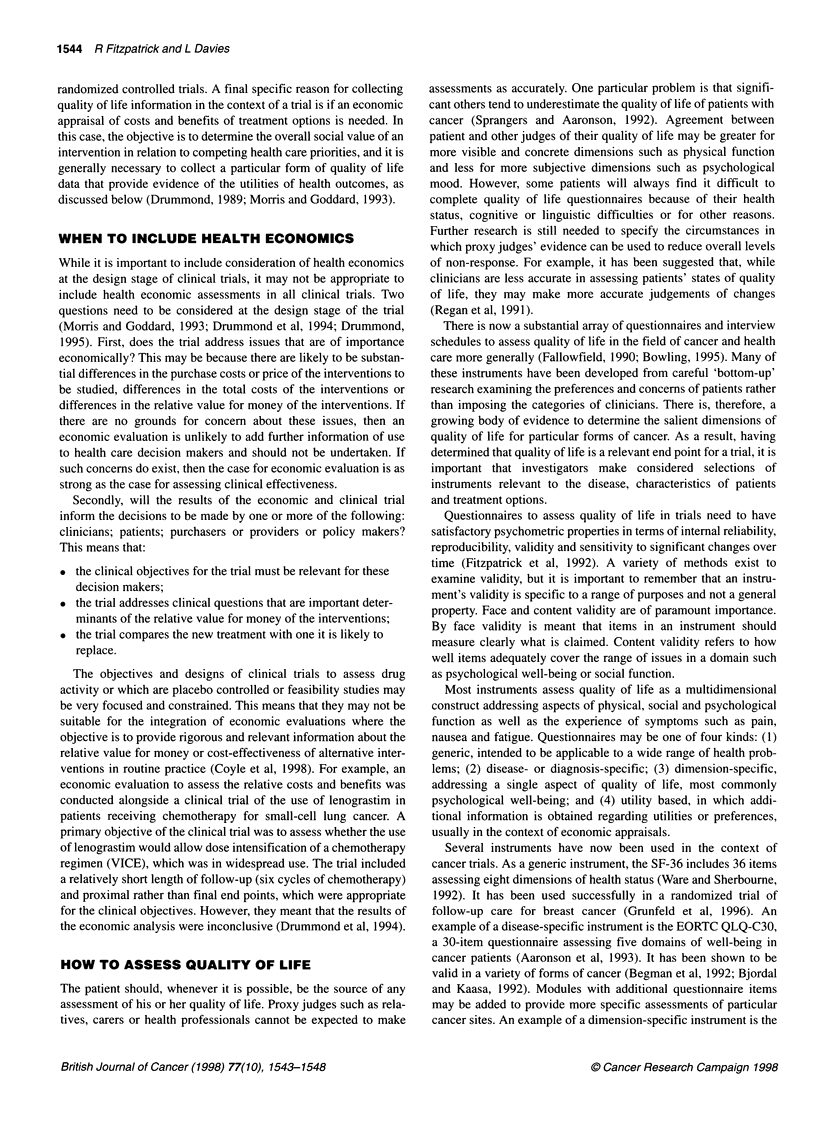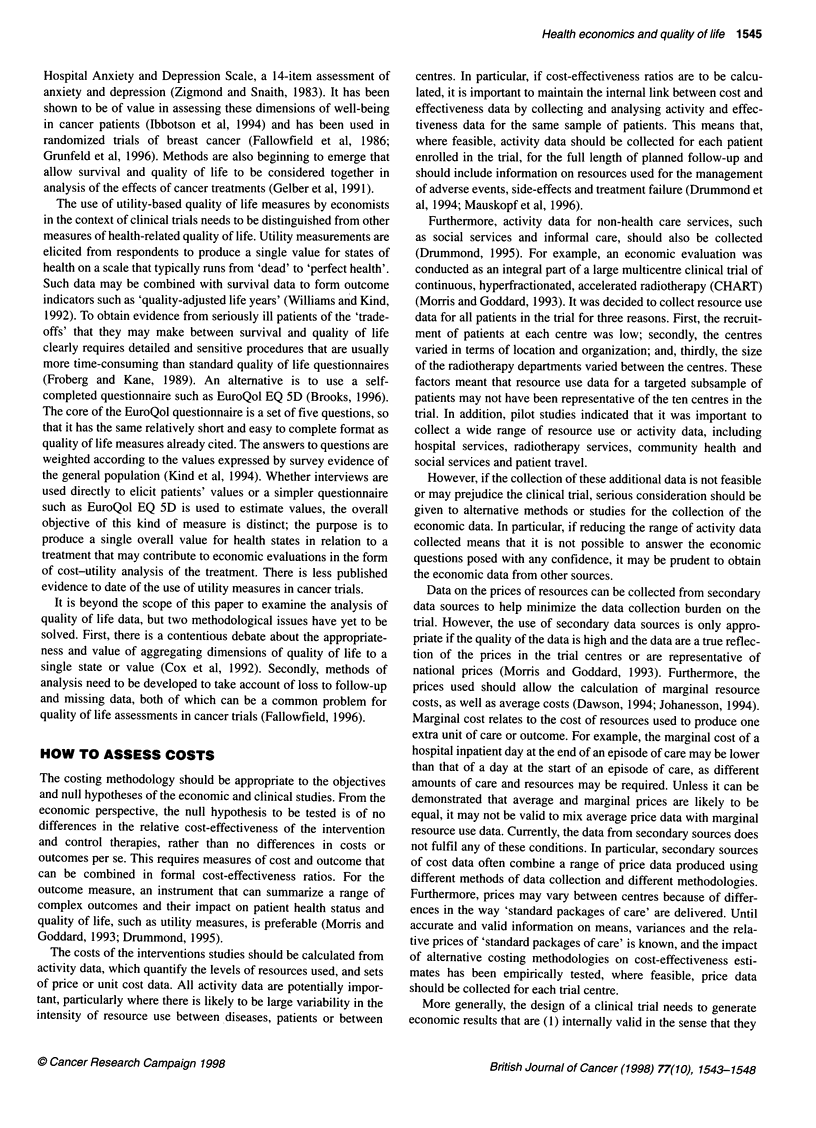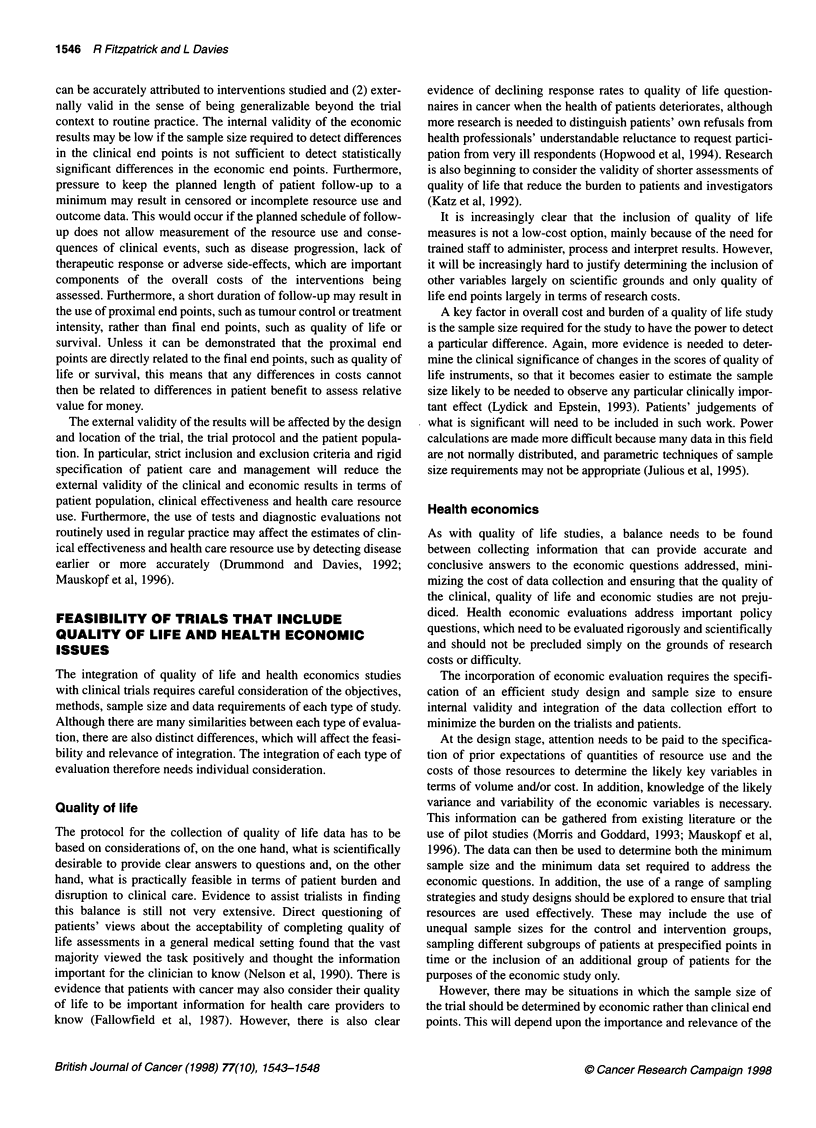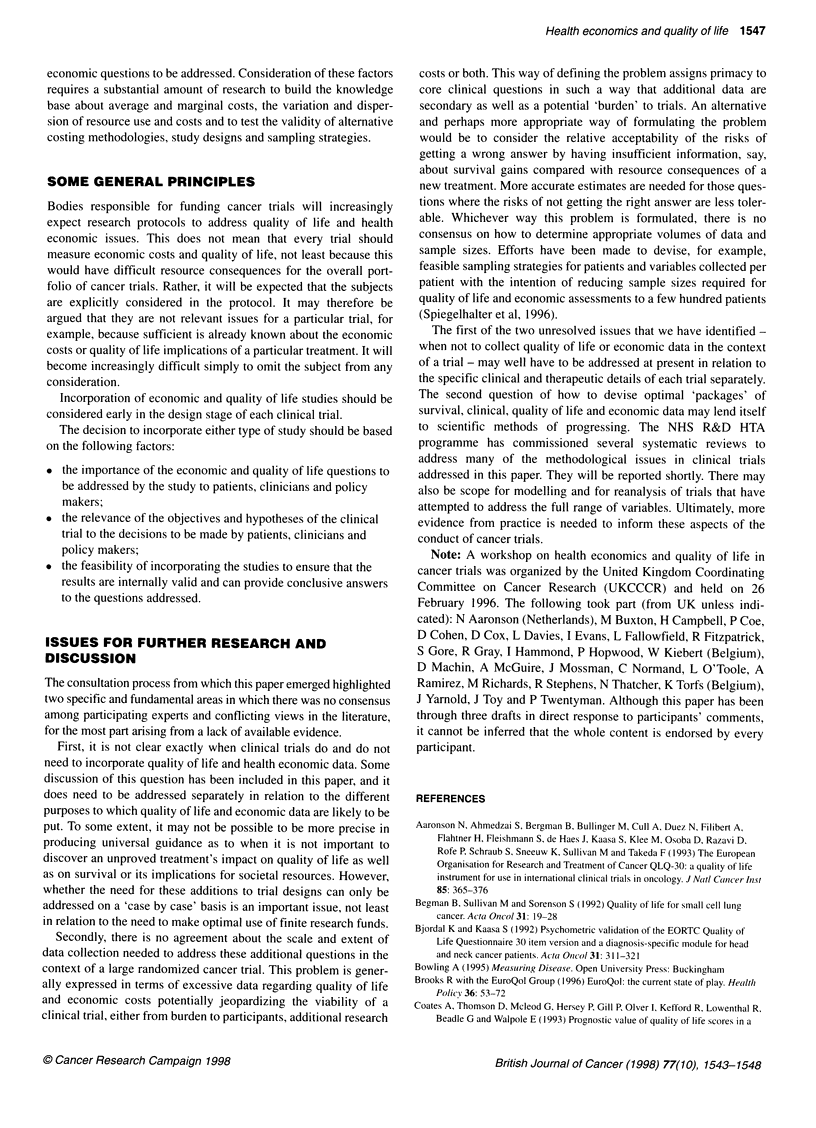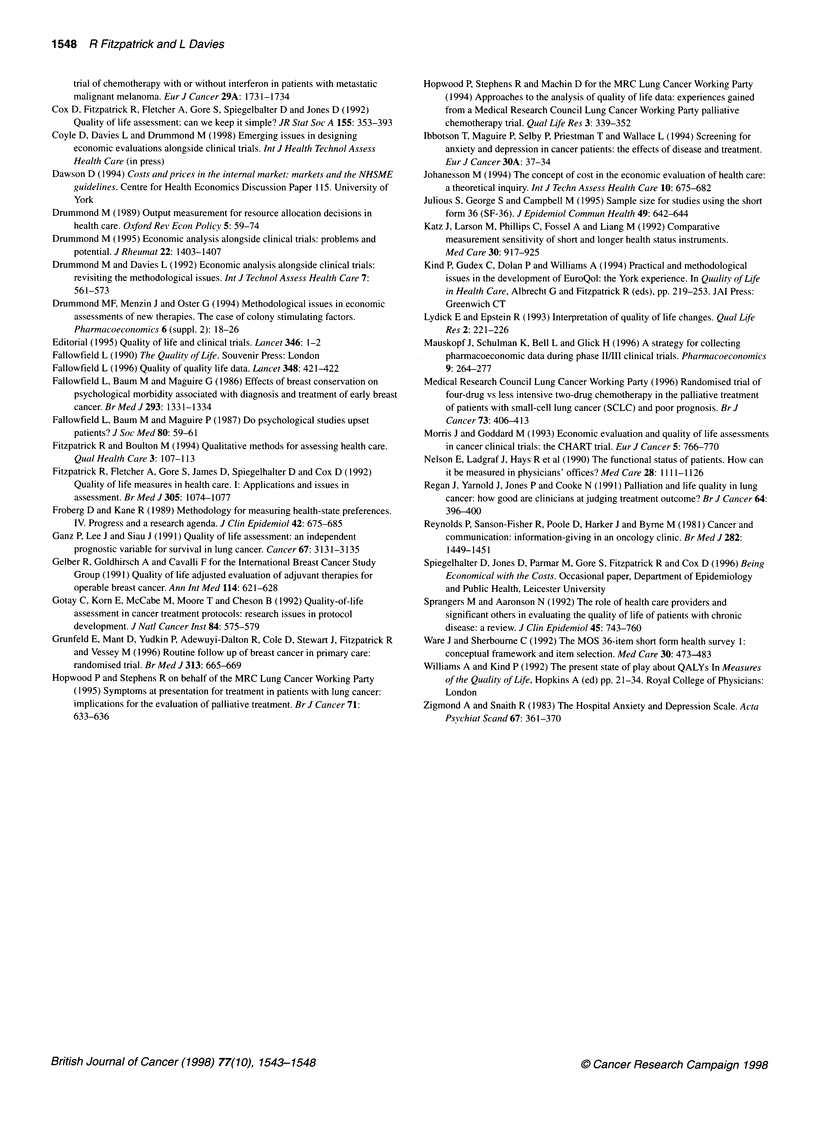# Health economics and quality of life in cancer trials: report based on a UKCCCR workshop. United Kingdom Coordinating Committee on Cancer Research.

**DOI:** 10.1038/bjc.1998.254

**Published:** 1998-05

**Authors:** R. Fitzpatrick, L. Davies

**Affiliations:** Division of Public Health and Primary Health Care, Institute of Health Sciences, University of Oxford, Headington, UK.


British Journal of Cancer (1998) 77(10), 1543–1548
© 1998 Cancer Research Campaign

### Health economics and quality of life in cancer trials: report based on a UKCCCR workshop

**R Fitzpatrick[1] and L Davies[2]**

[1]Division of Public Health and Primary Health Care, Institute of Health Sciences, University of Oxford, Headington OX3 7LF, UK; [2]Centre for Health Economics, University of York, Heslington, York YO1 5DD, UK

There is increasing pressure to incorporate health economics and quality of life assessment into clinical trials of interventions for the treatment of cancer. This pressure has arisen from the interests of agencies funding research (such as the MRC, the NHS R&D programme and the pharmaceutical industry) and the consumers of research information. These include local and national clinical decision makers, regulatory and reimbursement agencies and patients. However, the integration of quality of life and health economic assessments into the clinical trial process is not straightforward and has raised several issues of concern to all involved in health service and clinical research. Broadly speaking, there are three main sources of concern. First, there are uncertainties as to when to include such evaluations in clinical trials. Secondly, there are concerns about methodology to ensure the validity of health economics and quality of life aspects of clinical trial design. Thirdly, the increasing size required for clinical trials has led to concerns about the feasibility of trials and the willingness of clinicians and patients to participate if substantial additional data are collected. A major issue is to ensure that the inclusion of health economics and quality of life assessment does not prejudice long-term patient health by inefficient evaluation of new and existing interventions.

The issues of quality of life and health economics are often considered together in the context of clinical trials and, indeed, some types of economic appraisal may use particular kinds of quality of life measures to assess benefits in relation to costs. However, overall it is important to recognize that, to a considerable extent, quality of life and economic data address distinct questions. For the most part, they are therefore examined separately in this paper.

This paper has emerged from what may be considered an informal 'nominal group' technique, whereby the extent of consensus on a complex question is assessed (Fitzpatrick and Boulton, 1994). The UKCCCR held a 1-day workshop to bring together clinicians, health economists, experts in quality of life evaluation, statisticians and health care purchasers to discuss the role of health economics and quality of life in cancer trials and to make recommendations to the Trials Committee and the Main Committee of the UKCCCR. The authors of this paper were invited to summarize the discussions that occurred at the meeting. This took the form of an earlier draft of the current paper, which was then circulated to individuals who had attended. Participants were invited to send in written comments, which were used to revise the paper. A revised version was also circulated and again modified in response to additional comments. Because there was no basic agreement on two major issues, it seemed more accurate and informative to report these two points at the end of this paper as issues requiring further research and, possibly, more focused attention in further discussions. These two unresolved issues are (1) defining occasions when quality of life and economic data are not needed in trials and (2) defining the scale and extent of data collection required to address quality of life and economic questions in trials.

*Received 28 October 1997*
*Accepted 17 November 1997*

*Correspondence to:* R Fitzpatrick

### WHEN TO INCLUDE QUALITY OF LIFE

It is increasingly recognized that interventions for cancer are concerned with the quality of life of patients as well as survival. Clinical trials will also therefore increasingly address such end points. However, as with any other element in a clinical trial, it is essential that quality of life measures are included because they address an important question in a relevant way, rather than as a ritualistic feature to placate funding bodies or other parties. There are useful discussions of the kinds of trials in which quality of life is particularly relevant (Gotay et al, 1992; Editorial, *Lancet* 1995). Clearly, quality of life is the primary end point for many interventions such as palliative treatment. It is also critical where survival is thought likely to be equivalent between arms of a trial, for example when a more conservative treatment is being evaluated. Quality of life is also a salient issue in a trial in which survival gains of a new treatment may be quite small and potentially offset by deterioration in current quality of life. For the same reasons that treatment effects upon survival are harder to determine in non-randomized designs or in studies that are statistically underpowered, so too, where quality of life is a clinical end point, it is unlikely to be appropriate to 'bolt on' small-scale descriptive studies to address outcomes of a treatment adequately in terms of quality of life.

There are broader reasons that may justify the inclusion of quality of life measures in clinical trials. Clinicians increasingly face requests for information from patients about the likely course of their illness or the consequences of treatment, answers to which can only be given accurately if such evidence has been gathered systematically (Reynolds et al, 1981). There is also some evidence that quality of life data may be of prognostic value (Ganz et al, 1991; Coates et al, 1993). Randomized controlled trials incorporating quality of life outcome measures have provided invaluable information for such purposes (Hopwood and Stephens, 1995; MRC Lung Cancer Working Party, 1996). However, these broader objectives for increased understanding of quality of life in cancer can be pursued in the context of observational studies as well as randomized controlled trials. A final specific reason for collecting quality of life information in the context of a trial is if an economic appraisal of costs and benefits of treatment options is needed. In this case, the objective is to determine the overall social value of an intervention in relation to competing health care priorities, and it is generally necessary to collect a particular form of quality of life data that provide evidence of the utilities of health outcomes, as discussed below (Drummond, 1989; Morris and Goddard, 1993).

### WHEN TO INCLUDE HEALTH ECONOMICS

While it is important to include consideration of health economics at the design stage of clinical trials, it may not be appropriate to include health economic assessments in all clinical trials. Two questions need to be considered at the design stage of the trial (Morris and Goddard, 1993; Drummond et al, 1994; Drummond, 1995). First, does the trial address issues that are of importance economically? This may be because there are likely to be substantial differences in the purchase costs or price of the interventions to be studied, differences in the total costs of the interventions or differences in the relative value for money of the interventions. If there are no grounds for concern about these issues, then an economic evaluation is unlikely to add further information of use to health care decision makers and should not be undertaken. If such concerns do exist, then the case for economic evaluation is as strong as the case for assessing clinical effectiveness.

Secondly, will the results of the economic and clinical trial inform the decisions to be made by one or more of the following: clinicians; patients; purchasers or providers or policy makers? This means that:

- the clinical objectives for the trial must be relevant for these decision makers;
- the trial addresses clinical questions that are important determinants of the relative value for money of the interventions;
- the trial compares the new treatment with one it is likely to replace.

The objectives and designs of clinical trials to assess drug activity or which are placebo controlled or feasibility studies may be very focused and constrained. This means that they may not be suitable for the integration of economic evaluations where the objective is to provide rigorous and relevant information about the relative value for money or cost-effectiveness of alternative interventions in routine practice (Coyle et al, 1998). For example, an economic evaluation to assess the relative costs and benefits was conducted alongside a clinical trial of the use of lenograstim in patients receiving chemotherapy for small-cell lung cancer. A primary objective of the clinical trial was to assess whether the use of lenograstim would allow dose intensification of a chemotherapy regimen (VICE), which was in widespread use. The trial included a relatively short length of follow-up (six cycles of chemotherapy) and proximal rather than final end points, which were appropriate for the clinical objectives. However, they meant that the results of the economic analysis were inconclusive (Drummond et al, 1994).

### HOW TO ASSESS QUALITY OF LIFE

The patient should, whenever it is possible, be the source of any assessment of his or her quality of life. Proxy judges such as relatives, carers or health professionals cannot be expected to make assessments as accurately. One particular problem is that significant others tend to underestimate the quality of life of patients with cancer (Sprangers and Aaronson, 1992). Agreement between patient and other judges of their quality of life may be greater for more visible and concrete dimensions such as physical function and less for more subjective dimensions such as psychological mood. However, some patients will always find it difficult to complete quality of life questionnaires because of their health status, cognitive or linguistic difficulties or for other reasons. Further research is still needed to specify the circumstances in which proxy judges' evidence can be used to reduce overall levels of non-response. For example, it has been suggested that, while clinicians are less accurate in assessing patients' states of quality of life, they may make more accurate judgements of changes (Regan et al, 1991).

There is now a substantial array of questionnaires and interview schedules to assess quality of life in the field of cancer and health care more generally (Fallowfield, 1990; Bowling, 1995). Many of these instruments have been developed from careful 'bottom-up' research examining the preferences and concerns of patients rather than imposing the categories of clinicians. There is, therefore, a growing body of evidence to determine the salient dimensions of quality of life for particular forms of cancer. As a result, having determined that quality of life is a relevant end point for a trial, it is important that investigators make considered selections of instruments relevant to the disease, characteristics of patients and treatment options.

Questionnaires to assess quality of life in trials need to have satisfactory psychometric properties in terms of internal reliability, reproducibility, validity and sensitivity to significant changes over time (Fitzpatrick et al, 1992). A variety of methods exist to examine validity, but it is important to remember that an instrument's validity is specific to a range of purposes and not a general property. Face and content validity are of paramount importance. By face validity is meant that items in an instrument should measure clearly what is claimed. Content validity refers to how well items adequately cover the range of issues in a domain such as psychological well-being or social function.

Most instruments assess quality of life as a multidimensional construct addressing aspects of physical, social and psychological function as well as the experience of symptoms such as pain, nausea and fatigue. Questionnaires may be one of four kinds: (1) generic, intended to be applicable to a wide range of health problems; (2) disease- or diagnosis-specific; (3) dimension-specific, addressing a single aspect of quality of life, most commonly psychological well-being; and (4) utility based, in which additional information is obtained regarding utilities or preferences, usually in the context of economic appraisals.

Several instruments have now been used in the context of cancer trials. As a generic instrument, the SF-36 includes 36 items assessing eight dimensions of health status (Ware and Sherbourne, 1992). It has been used successfully in a randomized trial of follow-up care for breast cancer (Grunfeld et al, 1996). An example of a disease-specific instrument is the EORTC QLQ-C30, a 30-item questionnaire assessing five domains of well-being in cancer patients (Aaronson et al, 1993). It has been shown to be valid in a variety of forms of cancer (Begman et al, 1992; Bjordal and Kaasa, 1992). Modules with additional questionnaire items may be added to provide more specific assessments of particular cancer sites. An example of a dimension-specific instrument is the

© Cancer Research Campaign 1998

Hospital Anxiety and Depression Scale, a 14-item assessment of anxiety and depression (Zigmond and Snaith, 1983). It has been shown to be of value in assessing these dimensions of well-being in cancer patients (Ibbotson et al, 1994) and has been used in randomized trials of breast cancer (Fallowfield et al, 1986; Grunfeld et al, 1996). Methods are also beginning to emerge that allow survival and quality of life to be considered together in analysis of the effects of cancer treatments (Gelber et al, 1991).

The use of utility-based quality of life measures by economists in the context of clinical trials needs to be distinguished from other measures of health-related quality of life. Utility measurements are elicited from respondents to produce a single value for states of health on a scale that typically runs from 'dead' to 'perfect health'. Such data may be combined with survival data to form outcome indicators such as 'quality-adjusted life years' (Williams and Kind, 1992). To obtain evidence from seriously ill patients of the 'trade-offs' that they may make between survival and quality of life clearly requires detailed and sensitive procedures that are usually more time-consuming than standard quality of life questionnaires (Froberg and Kane, 1989). An alternative is to use a self-completed questionnaire such as EuroQol EQ 5D (Brooks, 1996). The core of the EuroQol questionnaire is a set of five questions, so that it has the same relatively short and easy to complete format as quality of life measures already cited. The answers to questions are weighted according to the values expressed by survey evidence of the general population (Kind et al, 1994). Whether interviews are used directly to elicit patients' values or a simpler questionnaire such as EuroQol EQ 5D is used to estimate values, the overall objective of this kind of measure is distinct; the purpose is to produce a single overall value for health states in relation to a treatment that may contribute to economic evaluations in the form of cost–utility analysis of the treatment. There is less published evidence to date of the use of utility measures in cancer trials.

It is beyond the scope of this paper to examine the analysis of quality of life data, but two methodological issues have yet to be solved. First, there is a contentious debate about the appropriateness and value of aggregating dimensions of quality of life to a single state or value (Cox et al, 1992). Secondly, methods of analysis need to be developed to take account of loss to follow-up and missing data, both of which can be a common problem for quality of life assessments in cancer trials (Fallowfield, 1996).

### HOW TO ASSESS COSTS

The costing methodology should be appropriate to the objectives and null hypotheses of the economic and clinical studies. From the economic perspective, the null hypothesis to be tested is of no differences in the relative cost-effectiveness of the intervention and control therapies, rather than no differences in costs or outcomes per se. This requires measures of cost and outcome that can be combined in formal cost-effectiveness ratios. For the outcome measure, an instrument that can summarize a range of complex outcomes and their impact on patient health status and quality of life, such as utility measures, is preferable (Morris and Goddard, 1993; Drummond, 1995).

The costs of the interventions studies should be calculated from activity data, which quantify the levels of resources used, and sets of price or unit cost data. All activity data are potentially important, particularly where there is likely to be large variability in the intensity of resource use between diseases, patients or between centres. In particular, if cost-effectiveness ratios are to be calculated, it is important to maintain the internal link between cost and effectiveness data by collecting and analysing activity and effectiveness data for the same sample of patients. This means that, where feasible, activity data should be collected for each patient enrolled in the trial, for the full length of planned follow-up and should include information on resources used for the management of adverse events, side-effects and treatment failure (Drummond et al, 1994; Mauskopf et al, 1996).

Furthermore, activity data for non-health care services, such as social services and informal care, should also be collected (Drummond, 1995). For example, an economic evaluation was conducted as an integral part of a large multicentre clinical trial of continuous, hyperfractionated, accelerated radiotherapy (CHART) (Morris and Goddard, 1993). It was decided to collect resource use data for all patients in the trial for three reasons. First, the recruitment of patients at each centre was low; secondly, the centres varied in terms of location and organization; and, thirdly, the size of the radiotherapy departments varied between the centres. These factors meant that resource use data for a targeted subsample of patients may not have been representative of the ten centres in the trial. In addition, pilot studies indicated that it was important to collect a wide range of resource use or activity data, including hospital services, radiotherapy services, community health and social services and patient travel.

However, if the collection of these additional data is not feasible or may prejudice the clinical trial, serious consideration should be given to alternative methods or studies for the collection of the economic data. In particular, if reducing the range of activity data collected means that it is not possible to answer the economic questions posed with any confidence, it may be prudent to obtain the economic data from other sources.

Data on the prices of resources can be collected from secondary data sources to help minimize the data collection burden on the trial. However, the use of secondary data sources is only appropriate if the quality of the data is high and the data are a true reflection of the prices in the trial centres or are representative of national prices (Morris and Goddard, 1993). Furthermore, the prices used should allow the calculation of marginal resource costs, as well as average costs (Dawson, 1994; Johanesson, 1994). Marginal cost relates to the cost of resources used to produce one extra unit of care or outcome. For example, the marginal cost of a hospital inpatient day at the end of an episode of care may be lower than that of a day at the start of an episode of care, as different amounts of care and resources may be required. Unless it can be demonstrated that average and marginal prices are likely to be equal, it may not be valid to mix average price data with marginal resource use data. Currently, the data from secondary sources does not fulfil any of these conditions. In particular, secondary sources of cost data often combine a range of price data produced using different methods of data collection and different methodologies. Furthermore, prices may vary between centres because of differences in the way 'standard packages of care' are delivered. Until accurate and valid information on means, variances and the relative prices of 'standard packages of care' is known, and the impact of alternative costing methodologies on cost-effectiveness estimates has been empirically tested, where feasible, price data should be collected for each trial centre.

More generally, the design of a clinical trial needs to generate economic results that are (1) internally valid in the sense that they

© Cancer Research Campaign 1998

can be accurately attributed to interventions studied and (2) externally valid in the sense of being generalizable beyond the trial context to routine practice. The internal validity of the economic results may be low if the sample size required to detect differences in the clinical end points is not sufficient to detect statistically significant differences in the economic end points. Furthermore, pressure to keep the planned length of patient follow-up to a minimum may result in censored or incomplete resource use and outcome data. This would occur if the planned schedule of follow-up does not allow measurement of the resource use and consequences of clinical events, such as disease progression, lack of therapeutic response or adverse side-effects, which are important components of the overall costs of the interventions being assessed. Furthermore, a short duration of follow-up may result in the use of proximal end points, such as tumour control or treatment intensity, rather than final end points, such as quality of life or survival. Unless it can be demonstrated that the proximal end points are directly related to the final end points, such as quality of life or survival, this means that any differences in costs cannot then be related to differences in patient benefit to assess relative value for money.

The external validity of the results will be affected by the design and location of the trial, the trial protocol and the patient population. In particular, strict inclusion and exclusion criteria and rigid specification of patient care and management will reduce the external validity of the clinical and economic results in terms of patient population, clinical effectiveness and health care resource use. Furthermore, the use of tests and diagnostic evaluations not routinely used in regular practice may affect the estimates of clinical effectiveness and health care resource use by detecting disease earlier or more accurately (Drummond and Davies, 1992; Mauskopf et al, 1996).

### FEASIBILITY OF TRIALS THAT INCLUDE QUALITY OF LIFE AND HEALTH ECONOMIC ISSUES

The integration of quality of life and health economics studies with clinical trials requires careful consideration of the objectives, methods, sample size and data requirements of each type of study. Although there are many similarities between each type of evaluation, there are also distinct differences, which will affect the feasibility and relevance of integration. The integration of each type of evaluation therefore needs individual consideration.

### Quality of life

The protocol for the collection of quality of life data has to be based on considerations of, on the one hand, what is scientifically desirable to provide clear answers to questions and, on the other hand, what is practically feasible in terms of patient burden and disruption to clinical care. Evidence to assist trialists in finding this balance is still not very extensive. Direct questioning of patients' views about the acceptability of completing quality of life assessments in a general medical setting found that the vast majority viewed the task positively and thought the information important for the clinician to know (Nelson et al, 1990). There is evidence that patients with cancer may also consider their quality of life to be important information for health care providers to know (Fallowfield et al, 1987). However, there is also clear evidence of declining response rates to quality of life questionnaires in cancer when the health of patients deteriorates, although more research is needed to distinguish patients' own refusals from health professionals' understandable reluctance to request participation from very ill respondents (Hopwood et al, 1994). Research is also beginning to consider the validity of shorter assessments of quality of life that reduce the burden to patients and investigators (Katz et al, 1992).

It is increasingly clear that the inclusion of quality of life measures is not a low-cost option, mainly because of the need for trained staff to administer, process and interpret results. However, it will be increasingly hard to justify determining the inclusion of other variables largely on scientific grounds and only quality of life end points largely in terms of research costs.

A key factor in overall cost and burden of a quality of life study is the sample size required for the study to have the power to detect a particular difference. Again, more evidence is needed to determine the clinical significance of changes in the scores of quality of life instruments, so that it becomes easier to estimate the sample size likely to be needed to observe any particular clinically important effect (Lydick and Epstein, 1993). Patients' judgements of what is significant will need to be included in such work. Power calculations are made more difficult because many data in this field are not normally distributed, and parametric techniques of sample size requirements may not be appropriate (Julious et al, 1995).

### Health economics

As with quality of life studies, a balance needs to be found between collecting information that can provide accurate and conclusive answers to the economic questions addressed, minimizing the cost of data collection and ensuring that the quality of the clinical, quality of life and economic studies are not prejudiced. Health economic evaluations address important policy questions, which need to be evaluated rigorously and scientifically and should not be precluded simply on the grounds of research costs or difficulty.

The incorporation of economic evaluation requires the specification of an efficient study design and sample size to ensure internal validity and integration of the data collection effort to minimize the burden on the trialists and patients.

At the design stage, attention needs to be paid to the specification of prior expectations of quantities of resource use and the costs of those resources to determine the likely key variables in terms of volume and/or cost. In addition, knowledge of the likely variance and variability of the economic variables is necessary. This information can be gathered from existing literature or the use of pilot studies (Morris and Goddard, 1993; Mauskopf et al, 1996). The data can then be used to determine both the minimum sample size and the minimum data set required to address the economic questions. In addition, the use of a range of sampling strategies and study designs should be explored to ensure that trial resources are used effectively. These may include the use of unequal sample sizes for the control and intervention groups, sampling different subgroups of patients at prespecified points in time or the inclusion of an additional group of patients for the purposes of the economic study only.

However, there may be situations in which the sample size of the trial should be determined by economic rather than clinical end points. This will depend upon the importance and relevance of the

© Cancer Research Campaign 1998

economic questions to be addressed. Consideration of these factors requires a substantial amount of research to build the knowledge base about average and marginal costs, the variation and dispersion of resource use and costs and to test the validity of alternative costing methodologies, study designs and sampling strategies.

### SOME GENERAL PRINCIPLES

Bodies responsible for funding cancer trials will increasingly expect research protocols to address quality of life and health economic issues. This does not mean that every trial should measure economic costs and quality of life, not least because this would have difficult resource consequences for the overall portfolio of cancer trials. Rather, it will be expected that the subjects are explicitly considered in the protocol. It may therefore be argued that they are not relevant issues for a particular trial, for example, because sufficient is already known about the economic costs or quality of life implications of a particular treatment. It will become increasingly difficult simply to omit the subject from any consideration.

Incorporation of economic and quality of life studies should be considered early in the design stage of each clinical trial.

The decision to incorporate either type of study should be based on the following factors:

- the importance of the economic and quality of life questions to be addressed by the study to patients, clinicians and policy makers;
- the relevance of the objectives and hypotheses of the clinical trial to the decisions to be made by patients, clinicians and policy makers;
- the feasibility of incorporating the studies to ensure that the results are internally valid and can provide conclusive answers to the questions addressed.

### ISSUES FOR FURTHER RESEARCH AND DISCUSSION

The consultation process from which this paper emerged highlighted two specific and fundamental areas in which there was no consensus among participating experts and conflicting views in the literature, for the most part arising from a lack of available evidence.

First, it is not clear exactly when clinical trials do and do not need to incorporate quality of life and health economic data. Some discussion of this question has been included in this paper, and it does need to be addressed separately in relation to the different purposes to which quality of life and economic data are likely to be put. To some extent, it may not be possible to be more precise in producing universal guidance as to when it is not important to discover an unproved treatment's impact on quality of life as well as on survival or its implications for societal resources. However, whether the need for these additions to trial designs can only be addressed on a 'case by case' basis is an important issue, not least in relation to the need to make optimal use of finite research funds.

Secondly, there is no agreement about the scale and extent of data collection needed to address these additional questions in the context of a large randomized cancer trial. This problem is generally expressed in terms of excessive data regarding quality of life and economic costs potentially jeopardizing the viability of a clinical trial, either from burden to participants, additional research costs or both. This way of defining the problem assigns primacy to core clinical questions in such a way that additional data are secondary as well as a potential 'burden' to trials. An alternative and perhaps more appropriate way of formulating the problem would be to consider the relative acceptability of the risks of getting a wrong answer by having insufficient information, say, about survival gains compared with resource consequences of a new treatment. More accurate estimates are needed for those questions where the risks of not getting the right answer are less tolerable. Whichever way this problem is formulated, there is no consensus on how to determine appropriate volumes of data and sample sizes. Efforts have been made to devise, for example, feasible sampling strategies for patients and variables collected per patient with the intention of reducing sample sizes required for quality of life and economic assessments to a few hundred patients (Spiegelhalter et al, 1996).

The first of the two unresolved issues that we have identified – when not to collect quality of life or economic data in the context of a trial – may well have to be addressed at present in relation to the specific clinical and therapeutic details of each trial separately. The second question of how to devise optimal 'packages' of survival, clinical, quality of life and economic data may lend itself to scientific methods of progressing. The NHS R&D HTA programme has commissioned several systematic reviews to address many of the methodological issues in clinical trials addressed in this paper. They will be reported shortly. There may also be scope for modelling and for reanalysis of trials that have attempted to address the full range of variables. Ultimately, more evidence from practice is needed to inform these aspects of the conduct of cancer trials.

**Note:** A workshop on health economics and quality of life in cancer trials was organized by the United Kingdom Coordinating Committee on Cancer Research (UKCCCR) and held on 26 February 1996. The following took part (from UK unless indicated): N Aaronson (Netherlands), M Buxton, H Campbell, P Coe, D Cohen, D Cox, L Davies, I Evans, L Fallowfield, R Fitzpatrick, S Gore, R Gray, I Hammond, P Hopwood, W Kiebert (Belgium), D Machin, A McGuire, J Mossman, C Normand, L O'Toole, A Ramirez, M Richards, R Stephens, N Thatcher, K Torfs (Belgium), J Yarnold, J Toy and P Twentyman. Although this paper has been through three drafts in direct response to participants' comments, it cannot be inferred that the whole content is endorsed by every participant.

### REFERENCES

Aaronson N, Ahmedzai S, Bergman B, Bullinger M, Cull A, Duez N, Filibert A, Flahtner H, Fleishmann S, de Haes J, Kaasa S, Klee M, Osoba D, Razavi D, Rofe P, Schraub S, Sneeuw K, Sullivan M and Takeda F (1993) The European Organisation for Research and Treatment of Cancer QLQ-30: a quality of life instrument for use in international clinical trials in oncology. *J Natl Cancer Inst* **85**: 365–376

Begman B, Sullivan M and Sorenson S (1992) Quality of life for small cell lung cancer. *Acta Oncol* **31**: 19–28

Bjordal K and Kaasa S (1992) Psychometric validation of the EORTC Quality of Life Questionnaire 30 item version and a diagnosis-specific module for head and neck cancer patients. *Acta Oncol* **31**: 311–321

Bowling A (1995) *Measuring Disease*. Open University Press: Buckingham

Brooks R with the EuroQol Group (1996) EuroQol: the current state of play. *Health Policy* **36**: 53–72

Coates A, Thomson D, Mcleod G, Hersey P, Gill P, Olver I, Kefford R, Lowenthal R, Beadle G and Walpole E (1993) Prognostic value of quality of life scores in a

© Cancer Research Campaign 1998

trial of chemotherapy with or without interferon in patients with metastatic malignant melanoma. *Eur J Cancer* **29A**: 1731–1734

Cox D, Fitzpatrick R, Fletcher A, Gore S, Spiegelbalter D and Jones D (1992) Quality of life assessment: can we keep it simple? *JR Stat Soc A* **155**: 353–393

Coyle D, Davies L and Drummond M (1998) Emerging issues in designing economic evaluations alongside clinical trials. *Int J Health Technol Assess Health Care* (in press)

Dawson D (1994) *Costs and prices in the internal market: markets and the NHSME guidelines*. Centre for Health Economics Discussion Paper 115. University of York

Drummond M (1989) Output measurement for resource allocation decisions in health care. *Oxford Rev Econ Policy* **5**: 59–74

Drummond M (1995) Economic analysis alongside clinical trials: problems and potential. *J Rheumat* **22**: 1403–1407

Drummond M and Davies L (1992) Economic analysis alongside clinical trials: revisiting the methodological issues. *Int J Technol Assess Health Care* **7**: 561–573

Drummond MF, Menzin J and Oster G (1994) Methodological issues in economic assessments of new therapies. The case of colony stimulating factors. *Pharmacoeconomics* **6** (suppl. 2): 18–26

Editorial (1995) Quality of life and clinical trials. *Lancet* **346**: 1–2

Fallowfield L (1990) *The Quality of Life*. Souvenir Press: London

Fallowfield L (1996) Quality of quality life data. *Lancet* **348**: 421–422

Fallowfield L, Baum M and Maguire G (1986) Effects of breast conservation on psychological morbidity associated with diagnosis and treatment of early breast cancer. *Br Med J* **293**: 1331–1334

Fallowfield L, Baum M and Maguire P (1987) Do psychological studies upset patients? *J Soc Med* **80**: 59–61

Fitzpatrick R and Boulton M (1994) Qualitative methods for assessing health care. *Qual Health Care* **3**: 107–113

Fitzpatrick R, Fletcher A, Gore S, James D, Spiegelhalter D and Cox D (1992) Quality of life measures in health care. I: Applications and issues in assessment. *Br Med J* **305**: 1074–1077

Froberg D and Kane R (1989) Methodology for measuring health-state preferences. IV. Progress and a research agenda. *J Clin Epidemiol* **42**: 675–685

Ganz P, Lee J and Siau J (1991) Quality of life assessment: an independent prognostic variable for survival in lung cancer. *Cancer* **67**: 3131–3135

Gelber R, Goldhirsch A and Cavalli F for the International Breast Cancer Study Group (1991) Quality of life adjusted evaluation of adjuvant therapies for operable breast cancer. *Ann Int Med* **114**: 621–628

Gotay C, Korn E, McCabe M, Moore T and Cheson B (1992) Quality-of-life assessment in cancer treatment protocols: research issues in protocol development. *J Natl Cancer Inst* **84**: 575–579

Grunfeld E, Mant D, Yudkin P, Adewuyi-Dalton R, Cole D, Stewart J, Fitzpatrick R and Vessey M (1996) Routine follow up of breast cancer in primary care: randomised trial. *Br Med J* **313**: 665–669

Hopwood P and Stephens R on behalf of the MRC Lung Cancer Working Party (1995) Symptoms at presentation for treatment in patients with lung cancer: implications for the evaluation of palliative treatment. *Br J Cancer* **71**: 633–636

Hopwood P, Stephens R and Machin D for the MRC Lung Cancer Working Party (1994) Approaches to the analysis of quality of life data: experiences gained from a Medical Research Council Lung Cancer Working Party palliative chemotherapy trial. *Qual Life Res* **3**: 339–352

Ibbotson T, Maguire P, Selby P, Priestman T and Wallace L (1994) Screening for anxiety and depression in cancer patients: the effects of disease and treatment. *Eur J Cancer* **30A**: 37–34

Johanesson M (1994) The concept of cost in the economic evaluation of health care: a theoretical inquiry. *Int J Techn Assess Health Care* **10**: 675–682

Julious S, George S and Campbell M (1995) Sample size for studies using the short form 36 (SF-36). *J Epidemiol Commun Health* **49**: 642–644

Katz J, Larson M, Phillips C, Fossel A and Liang M (1992) Comparative measurement sensitivity of short and longer health status instruments. *Med Care* **30**: 917–925

Kind P, Gudex C, Dolan P and Williams A (1994) Practical and methodological issues in the development of EuroQol: the York experience. In *Quality of Life in Health Care*, Albrecht G and Fitzpatrick R (eds), pp. 219–253. JAI Press: Greenwich CT

Lydick E and Epstein R (1993) Interpretation of quality of life changes. *Qual Life Res* **2**: 221–226

Mauskopf J, Schulman K, Bell L and Glick H (1996) A strategy for collecting pharmacoeconomic data during phase II/III clinical trials. *Pharmacoeconomics* **9**: 264–277

Medical Research Council Lung Cancer Working Party (1996) Randomised trial of four-drug vs less intensive two-drug chemotherapy in the palliative treatment of patients with small-cell lung cancer (SCLC) and poor prognosis. *Br J Cancer* **73**: 406–413

Morris J and Goddard M (1993) Economic evaluation and quality of life assessments in cancer clinical trials: the CHART trial. *Eur J Cancer* **5**: 766–770

Nelson E, Ladgraf J, Hays R et al (1990) The functional status of patients. How can it be measured in physicians' offices? *Med Care* **28**: 1111–1126

Regan J, Yarnold J, Jones P and Cooke N (1991) Palliation and life quality in lung cancer: how good are clinicians at judging treatment outcome? *Br J Cancer* **64**: 396–400

Reynolds P, Sanson-Fisher R, Poole D, Harker J and Byrne M (1981) Cancer and communication: information-giving in an oncology clinic. *Br Med J* **282**: 1449–1451

Spiegelhalter D, Jones D, Parmar M, Gore S, Fitzpatrick R and Cox D (1996) *Being Economical with the Costs*. Occasional paper, Department of Epidemiology and Public Health, Leicester University

Sprangers M and Aaronson N (1992) The role of health care providers and significant others in evaluating the quality of life of patients with chronic disease: a review. *J Clin Epidemiol* **45**: 743–760

Ware J and Sherbourne C (1992) The MOS 36-item short form health survey 1: conceptual framework and item selection. *Med Care* **30**: 473–483

Williams A and Kind P (1992) The present state of play about QALYs In *Measures of the Quality of Life*, Hopkins A (ed) pp. 21–34. Royal College of Physicians: London

Zigmond A and Snaith R (1983) The Hospital Anxiety and Depression Scale. *Acta Psychiat Scand* **67**: 361–370

*© Cancer Research Campaign 1998*